# Rectal inflammatory myofibroblastic tumor: Case report and literature review

**DOI:** 10.1097/MD.0000000000027008

**Published:** 2021-08-20

**Authors:** Hyung-Hoon Oh, Young-Eun Joo

**Affiliations:** Department of Internal Medicine, Chonnam National University Medical School, Gwangju, Korea.

**Keywords:** inflammatory myofibroblastic tumor, rectum

## Abstract

**Rationale:**

Rectal inflammatory myofibroblastic tumor (IMT) is an extremely rare mesenchymal tumor characterized by a mixture of spindle-shaped myofibroblasts or fibroblasts and inflammatory infiltration of lymphocytes and plasma cells. To date, only 8 cases of rectal IMT have been reported. Herein, we report an additional case of rectal IMT in a 28-year-old woman.

**Patient concerns:**

A 28-year-old woman presented with abdominal pain and hematochezia.

**Diagnoses:**

Colonoscopy showed a 3.0-cm subepithelial tumor with central ulceration, covered by white exudate in the rectum. Rectal magnetic resonance imaging revealed a 4.0 × 3.0-cm-sized well-defined subepithelial tumor in the right wall of the rectum, with suspicious right perirectal fat infiltration.

**Interventions:**

Laparoscopic anterior resection was performed. Microscopic examination of the surgical specimen revealed bland-looking spindle cells intermingled with lymphoplasma cells. Immunohistochemistry and fluorescence in situ hybridization showed anaplastic lymphoma kinase positivity and anaplastic lymphoma kinase positivity rearrangement. Rectal IMT was confirmed based on histological, immunohistochemical, and fluorescence in situ hybridization findings. The patient was doing well without evidence of tumor recurrence 1 year after the surgery.

**Lessons:**

Rectal IMT, despite its rarity, should be considered in the differential diagnosis of rectal cancer. Second, an accurate histopathologic diagnosis and complete surgical resection can be the most important approaches to offer a chance for the cure of rectal IMT.

## 1 Introduction

Inflammatory myofibroblastic tumor (IMT) is a rare solid tumor characterized by the proliferation of spindle-shaped myofibroblasts and inflammatory infiltration of lymphocytes and plasma cells.^[1,2]^ Although it is most commonly found in the lung, it can occur in any part of the body and can be found in any age group of patients.^[3]^ The occurrence of IMT arising from the colorectum was first described by Coffin et al. in 1995.^[4]^ Subsequently, approximately 60 cases of colorectal IMT have been documented in the literature.^[5]^ The frequency of its occurrence was in the order of the ascending colon, transverse colon, cecum, sigmoid colon, descending colon, and rectum.^[5]^

Rectal IMT is extremely rare. To date, only 8 cases of rectal IMT have been reported.^[6–13]^ Herein, we report an additional case of rectal IMT in a 28-year-old woman and review the literature pertaining to this condition.

## 2 Case report

**Figure 1:**
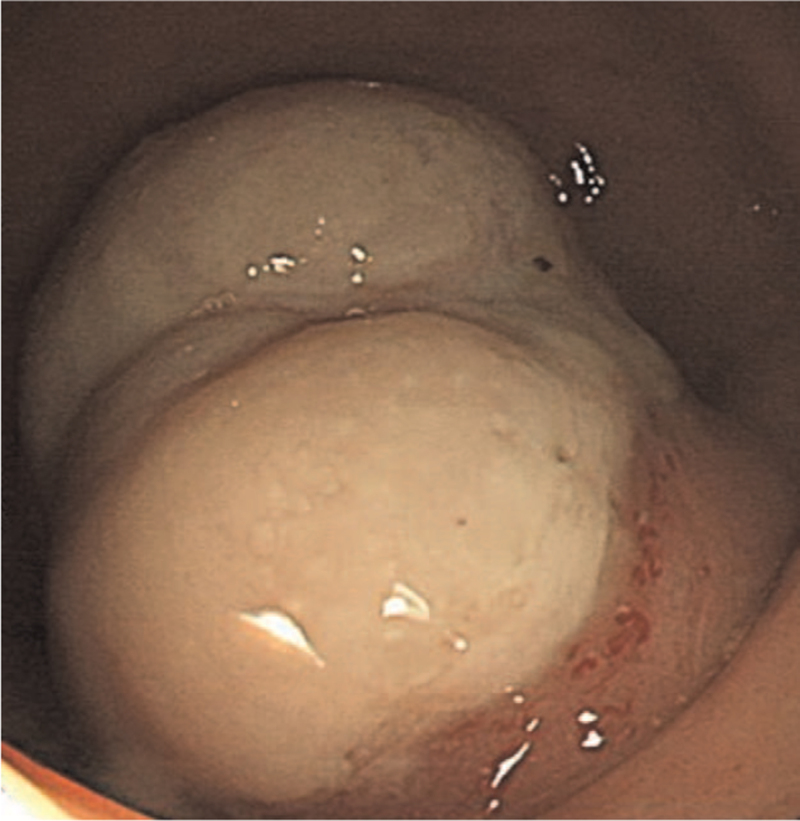
Colonoscopy shows a 3.0-cm-sized subepithelial tumor with central ulceration, covered by white exudate in the rectum, 9 cm above the anal verge.

**Figure 2:**
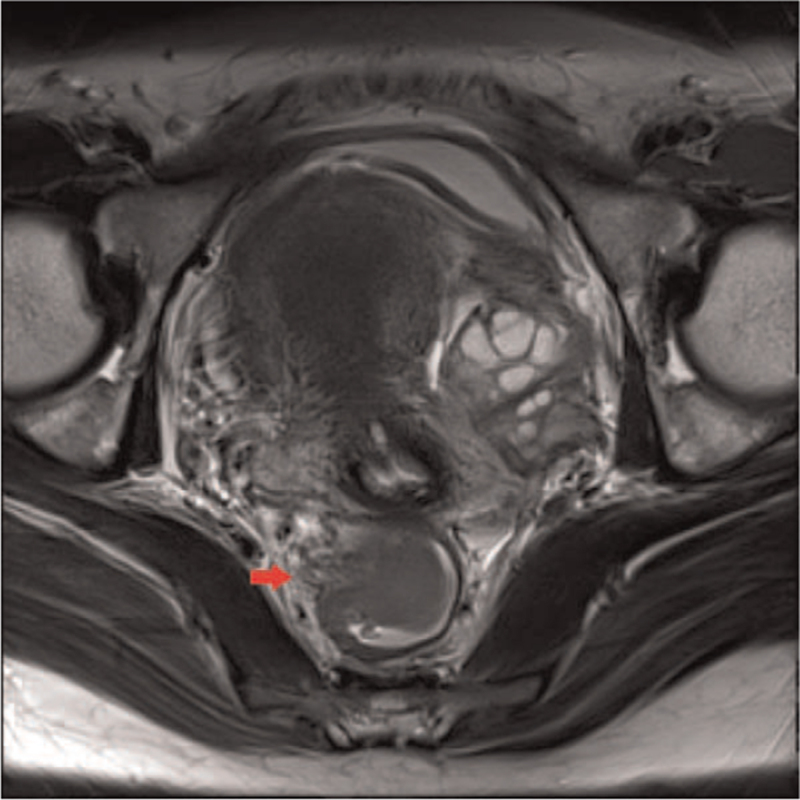
Rectal magnetic resonance imaging shows a 4.0 × 3.0-cm-sized well-defined intraluminal mass with intact overlying mucosa in the right wall of the rectum with suspicious right perirectal fat infiltration (red arrow).

**Figure 3:**
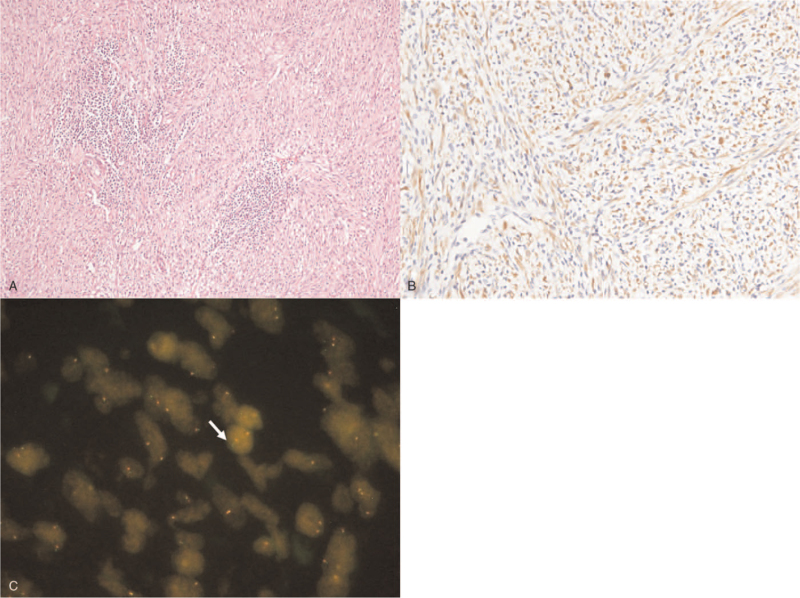
Microscopic findings of resected specimen. (A) Routine histology, with hematoxylin–eosin staining, shows the bland-looking spindle cells intermingled with lymphoplasma cells (×100). (B) The spindle cells are strongly positive for anaplastic lymphoma kinase (ALK) immunohistochemistry (×200). (C) Fluorescence in situ hybridization examination using the ALK break-apart probe demonstrates the presence of split red and green signals (white arrow), consistent with ALK rearrangement (×1000).

A 28-year-old woman was admitted to our hospital with a 1-month history of abdominal pain and hematochezia. She denied any previous medical history. On admission, her vital signs were normal. There was no abdominal tenderness or palpable mass on the abdomen, and other physical examinations were normal. Laboratory evaluation revealed a white blood cell count of 7000/mm^3^ (normal, 6000–10,000), hemoglobin level of 9.0 g/dL (normal, 12–16), mean corpuscular volume of 86.1 fL (normal, 80–99), mean corpuscular hemoglobin level of 28.3 pg (normal, 27–32), mean cell hemoglobin concentration of 32.9 g/dL (normal, 33–37), platelet count of 328,000/mm3 (normal, 130,000–450,000), serum albumin level of 4.1 g/dL (normal, 3.0–5.0), aspartate aminotransferase level of 29 U/L (normal, 5–37), alanine aminotransferase level of 40 U/L (normal, 5–40), alkaline phosphatase level of 88 U/L (normal, 39–117), and total bilirubin level of 0.4 mg/dL (normal, 0.35–1.3). The coagulation profiles were within normal limits. The C-reactive protein (CRP) level was increased to 8.67 mg/dL (normal, 0–0.3). Tumor markers including serum carcinoembryonic antigen, carbohydrate antigen (CA) 19-9, and CA 125 were all within normal limits. To determine the cause of hematochezia, we performed a colonoscopy. A 3.0-cm subepithelial tumor with central ulceration, covered by white exudate, was found in the rectum, 9 cm above the anal verge (Fig. 1). Colonoscopic biopsy of this lesion showed only mucopurulent necrotic tissue without malignant findings. To further evaluate this lesion, rectal magnetic resonance imaging and ^18^F-fluorodeoxyglucose positron emission tomography/computed tomography were performed. Rectal magnetic resonance imaging revealed a 4.0 × 3.0-cm-sized well-defined subepithelial tumor in the right wall of the rectum with suspicious right perirectal fat infiltration (Fig. 2A). In ^18^F-fluorodeoxyglucose positron emission tomography/computed tomography scan, a hypermetabolic lesion (20 standardized uptake values) was found in the rectum without regional or distant metastasis. We surgically removed the lesion for diagnostic and therapeutic purposes. Laparoscopic anterior resection was performed. Macroscopically, the specimen revealed a yellowish fungating mass with central ulceration covered with whitish mucus materials. Microscopic examination of the surgical specimen revealed bland-looking spindle cells intermingled with lymphoplasma cells (Fig. 3A). The spindle cells were strongly positive for anaplastic lymphoma kinase (ALK) immunohistochemistry (Fig. 3B), and fluorescence in situ hybridization examination using the ALK break-apart probe demonstrated the presence of split red and green signals, consistent with ALK rearrangement (Fig. 3C). Additional immunohistochemical staining showed that most cells were positive for actin and S-100 and negative for CD34 and C-kit. IMT involving the rectum was confirmed based on histological, immunohistochemical, and fluorescence in situ hybridization findings. She had been doing well without evidence of tumor recurrence in follow-up colonoscopy 1 year after the surgery. Informed consent was obtained from the patient for the purpose of publication.

## 3 Discussion

IMT is a rare mesenchymal tumor of intermediate malignant potential that occurs primarily in children and young adults and is commonly seen in the lungs, bladder, brain, mesentery, liver, and spleen. However, it may occur in diverse anatomical locations of the body, but rarely in the colorectum.^[1,3]^ To date, approximately 60 cases of colorectal IMT have been reported in the medical literature.^[5]^ The most common location of colorectal IMT is the ascending colon, followed by the transverse colon, cecum, sigmoid colon, descending colon, and rectum.^[5]^

Rectal IMT is extremely rare. In this report, a review of the medical literature disclosed 9 reported cases (including the present case) of IMT arising from the rectum (Table 1).^[6–13]^ IMT is found predominantly in children and young adults without a gender preference. In our study, patients with rectal IMTs were aged 1 to 81 (mean age, 28.7) years and included 5 men and 4 women.

**Table 1:** Summary of reported cases of inflammatory myofibroblastic tumor arising from rectum.

Patient no.	Author, year	Age (yr old)/sex	Colonoscopic description	Size (cm)	Endoscopic biopsy	ALK rearrangement	Presentation	Laboratory findings	Treatment	Follow-up	Recurrence or death
1	Sanders et al, 2001^[6]^	15/female	Large rectal mass	NA	Chronic inflammation	N/A	Abdominal pain, nausea, diarrhea, weight loss	Anemia, elevated ESR, CRP	Transanal resection	12 mo	No
2	Khoddami et al, 2006^[7]^	11/male	N/A	5.0 × 2.0	N/A	N/A	Hematochezia, fecal incontinence, abdominal pain, fatigue, weight loss	Microcytic hypochromic anemia, elevated ESR, CRP	Laparotomy	3 yr	No
3	Shi et al, 2010^[8]^	20/female	Elevated above the colorectal mucosa and involved the full thickness of the colorectal wall with ulceration of the luminal surface	4.3^a^	N/A	Positive	Abdominal pain, pelvic mass	N/A	Rectectomy	4 yr	No
4	Zhou et al, 2011^[9]^	13 mo/female	Hemispheroidal mass protruding from the anus with a white color and a broad pedicle	4.0 × 4.0 × 3.0	N/A	Positive	Abdominal mass	Anemia, elevated ESR, CRP, normal CEA, AFP, CA 19 to 9, CA 125, CA 15 to 3	Operation name not available	4.5 yr	No
5	Satahoo et al, 2013^[10]^	14/male	Shaggy white necrotic lesion with ulceration. Suspicious for rectal cancer with luminal stenosis	6.5^a^	Chronic inflammation with fibrosis without evidence of malignancy	Negative	Hematochezia, tenesmus, constipation, weight loss	Leukocytosis, elevated CRP, ESR, normal CEA, AFP	exploratory laparotomy with loop sigmoid colostomy	7 yr	No
6	Sun et al, 2014^[11]^	36/male	Soft tissue mass	3.0^a^	N/A	Positive	Hematochezia, tenesmus, constipation	N/A	Segmental resection. After recurrence, palliative resection, radiation therapy and chemotherapy with 2 courses of cisplatin and epirubicin. Celecoxib for 6 mo	20 mo	Recurrence at 18 mo. After reoperation no recurrence for 6 mo
7	Bai et al, 2020^[12]^	52/male	Suspicious of malignancy	8.0 × 5.0 × 4.0	Colonic mucosa and submucosa with ulceration, necrosis, hemorrhages and dense lymphoplasmacytic chronic inflammation	N/A	Abdominal pain, constipation, hematochezia, fecal incontinence, weight loss	Normal CEA	Sigmoid colostomy with antiviral treatment	N/A	No
8	Shimodaira et al, 2020^[13]^	81/male	2 cm sized ulcerated mass with sharply demarcated and raised margins with white exudate on the surface	2.2^a^	No malignant findings	Negative	Anal pain	N/A	Miles’ operation	1 mo	Died 1 mo after diagnosis (liver, bladder, prostate, pelvic floor metastasis)
9	This case	28/female	Subepithelial tumor covered by white exudate on the surface with central ulceration	4.0 × 3.0	Mucopurulent necrotic tissue without malignant findings	Positive	Abdominal pain, hematochezia	Anemia, elevated CRP, normal CEA, CA 19 to 9, CA to 125	Laparoscopic anterior resection	1 yr	No

AFP = alpha-fetoprotein, ALK = anaplastic lymphoma kinase, CA = carbohydrate antigen, CEA = carcinoembryonic antigen, CRP = C-reactive protein, ESR = erythrocyte sedimentation rate, N/A = not available.

a Only major axis available.

The presenting symptoms of rectal IMTs were abdominal pain (6 of 9 cases), hematochezia (5 of 9 cases), weight loss (4 of 9 cases), and constipation (3 of 9 cases). The common laboratory findings were elevated CRP level or erythrocyte sedimentation rate (5 of 9 cases) and anemia (4 of 9 cases). Tumor markers, including serum carcinoembryonic antigen, (CA) 19-9, and CA 125, were within normal limits (3 of 9 cases). Abdominal pain, hematochezia, elevation of CRP level or erythrocyte sedimentation rate, and anemia were the most common findings in rectal IMTs. The most common colonoscopic finding was a large rectal mass with central ulceration, similar to our case. These clinical and colonoscopic findings resemble those of rectal cancers. Therefore, the primary diagnosis of rectal IMT is difficult.

In our study, no cases were diagnosed as IMT with colonoscopic biopsy. However, the dramatic difference in the prognosis and treatment between rectal cancer and rectal IMT stresses the importance of differential diagnosis in rectal tumors. All cases, including ours, were diagnosed and treated with surgical resection. Therefore, surgery to obtain adequate tissue is necessary to achieve a satisfactory diagnosis and treatment.

IMT is a slow-growing, benign tumor with a potential risk of recurrence. Therefore, the World Health Organization has classified IMT as an intermediate neoplasm.^[14]^ The prognosis of IMT is good and is associated with distant metastasis in approximately 10% of cases. In our study, the mean follow-up time was 33.4 months (1 month–7 years). Two patients showed relapse during follow-up. One patient underwent reoperation,^[11]^ and 1 died of metastasis.^[13]^

ALK gene rearrangement on chromosome 2p23 is a well-known oncogenic driver in non-small cell lung cancer and has also been identified in approximately 50% of IMTs.^[3,15]^ Absent ALK gene rearrangement was associated with a higher overall age, subtle histologic differences, and death from disease or distant metastases, suggesting a favorable prognostic indicator.^[1,16,17]^ In our study, ALK gene rearrangement was detected in 4 cases, negative in 2 cases, and not evaluated in 3 cases. Among the 2 ALK-negative patients, 1 died of multiple metastases, including the liver, bladder, prostate, and pelvic floor. However, the role of ALK gene rearrangement as a prognostic indicator of rectal IMT remains unclear because of the limited number of patients with this disease.

## 4 Conclusion

Rectal IMT, despite its rarity, should be considered in the differential diagnosis of rectal cancer. Second, an accurate histopathologic diagnosis and complete surgical resection can be the most important approaches to offer a reasonable chance for the cure of rectal IMT.

## Author contributions

**Conceptualization:** Young-Eun Joo.

**Investigation:** Hyung-Hoon Oh.

**Resources:** Hyung-Hoon Oh, Young-Eun Joo.

**Supervision:** Young-Eun Joo.

**Writing – original draft:** Hyung-Hoon Oh, Young-Eun Joo.

**Writing – review & editing:** Hyung-Hoon Oh, Young Eun Joo.
